# Protein expression changes caused by spaceflight as measured for 18 Russian cosmonauts

**DOI:** 10.1038/s41598-017-08432-w

**Published:** 2017-08-15

**Authors:** Irina M. Larina, Andrew J. Percy, Juncong Yang, Christoph H. Borchers, Andrei M. Nosovsky, Anatoli I. Grigoriev, Evgeny N. Nikolaev

**Affiliations:** 1 Institute of Biomedical Problems, Russian Academy of Sciences, 76a Khoroshevskoye shosse, 123007 Moscow, Russia; 2 University of Victoria - Genome BC Proteomics Centre, University of Victoria, Vancouver Island Technology Park, 3101–4464 Markham Street, Victoria, British Columbia V8Z 7X8 Canada; 3 Department of Biochemistry and Microbiology, University of Victoria, Petch Building Room 207, 3800 Finnerty Road, Victoria, British Columbia V8P 5C2 Canada; 4 Lady Davis Institute, Jewish General Hospital, McGill University, 3755 Côte Ste-Catherine Road, Montreal, Quebec H3T 1E2 Canada; 5 Gerald Bronfman Department of Oncology, Jewish General Hospital, 3755 Côte Ste-Catherine Road, Montreal, Quebec H3T 1E2 Canada; 6 Skolkovo Institute of Science and Technology, Skoltech, Moscow region, Skolkovo village, Novaya st.100, 143025 Russia; 7 Moscow Institute of Physics and Technology, Moscow region, Dolgoprudnyj, Institutskij pr. 9, 141700 Russia; 8 Institute of Energy Problems of Chemical Physics Russian Academy of Sciences, 38 k.2 Leninskij pr., 119334 Moscow, Russia

**Keywords:** Protein-protein interaction networks, Predictive markers

## Abstract

The effects of spaceflight on human physiology is an increasingly studied field, yet the molecular mechanisms driving physiological changes remain unknown. With that in mind, this study was performed to obtain a deeper understanding of changes to the human proteome during space travel, by quantitating a panel of 125 proteins in the blood plasma of 18 Russian cosmonauts who had conducted long-duration missions to the International Space Station. The panel of labeled prototypic tryptic peptides from these proteins covered a concentration range of more than 5 orders of magnitude in human plasma. Quantitation was achieved by a well-established and highly-regarded targeted mass spectrometry approach involving multiple reaction monitoring in conjunction with stable isotope-labeled standards. Linear discriminant function analysis of the quantitative results revealed three distinct groups of proteins: 1) proteins with post-flight protein concentrations remaining stable, 2) proteins whose concentrations recovered slowly, or 3) proteins whose concentrations recovered rapidly to their pre-flight levels. Using a systems biology approach, nearly all of the reacting proteins could be linked to pathways that regulate the activities of proteases, natural immunity, lipid metabolism, coagulation cascades, or extracellular matrix metabolism.

## Introduction

The complex combination of factors that affect the human body during spaceflight are not among those which influenced human evolution on earth, so the human body is not necessarily adapted to them. The effect of space flight on humans has been studied by physiologists and medics for the past half-century^1–5^. Many changes have been observed on whole human organisms and on different physiological systems of cosmonauts after completion of space flight. In general, these systems constitute an aggregate of adaptive reactions involving all the functional systems and tissues of the organism. The principle effects of spaceflight factors include:

1. Energy imbalance - when the body’s energy expenditure is not reimbursed by the incoming food, which imposes serious consequences for many physical processes^2–4^
2. Negative balance of water and calcium^5, 6^, although sodium balance is possibly positive^7, 8^
3. Demineralization and modification of bone structure^9^
4. Ineffective thermal regulation^10, 11^
5. Shifts in biorhythms of heat production, hormonal secretion, and cardiac function^12–14^
6. Re-structuring of the vasomotion control^15^ and dysfunction of the vascular endothelium^16^
7. Muscular hypotrophy^17–19^, loss in tone and deterioration of force-velocity properties^20, 21^
8. Functional differentiation of the sensory systems and consequent disorders in motor control^21, 22^
9. Modification of pulmonary volumes, respiration biomechanics and chemoreceptor regulation^23, 24^
10. Immune system dysfunction^25–27^
11. Spaceflight anemia^28^.

The molecular mechanisms driving these changes remain unknown. Because proteins are key players in the adaptive processes in an organism, a panoramic picture of changes in protein expression may provide information about the mechanisms of adaptation.

In this mass spectromet﻿ry (MS)-﻿based study, quantitative proteomic analysis was performed on 54 plasma samples collected from 18 cosmonauts before and after long-duration spaceflights on the Russian module of the International Space Station (ISS). The duration of these flights was 158 ± 15 days, with the exception of one cosmonaut who flew for 429 days. The 142 extracellular proteins targeted in this study were not selected specifically for this particular space-oriented study but were a subset of proteins that were previously quantified in human plasma using multiple reaction monitoring (MRM)-MS with stable isotope-labeled peptides, and are reported to be putative biomarkers of non-communicable disease. Statistical interpretation of the subsequent results revealed three classes of proteins that could be grouped and linked to pathways involving metabolism and biochemical function. To the best of our knowledge, this study is the first relatively large-scale, MS-based proteomics investigation of the effects of space flight on plasma protein levels in cosmonauts’ blood, and it has yielded insights into the adaptive changes of this set of extracellular proteins.

## Methods

### Sample Collection

Whole blood samples were collected from 18 Russian cosmonauts (mean ± SD age: 44 ± 6 years old, all male) at 3 defined time points: 1) 30 days prior to launch (abbreviated L-30), 2) on the first day of recovery (R + 0), and 3) 7 days later (R + 7). The round-trip mission to the International Space Station (ISS) (430 km from launch point) took 6 hrs from launch to docking with the ISS and 3 hrs to return back from the ISS to earth. The first blood draw was performed 25.2 ± 0.1 hrs after landing. Blood was taken from a vein in the cubital fossa, collection was done in commercial SARSTEDT-Monovette^®^ tubes containing EDTA as the anticoagulant. After centrifugation for plasma separation (2000 rpm for 15 min, +4° C), the supernatant was frozen at −86 °C. No protease inhibitors or antimicrobial agents were added. All methods were performed in accordance with the relevant guidelines and regulations. All cosmonauts voluntarily completed an Informed Consent Form prior prior to sample donation. The described ISS-based experiments were approved by the Human Research Multilateral Review Board.

### Target Protein and Peptide Panel

The target panel for LC/MRM-MS interrogation comprised proteins for which assays had been developed previously^29^. The target proteins are classified as high-to-moderate abundance, spanning an approximate concentration range from 33 mg/mL to 44 ng/mL^29, 30^.

In the MRM-with-SIS-peptide quantitative approach, proteotypic peptides (usually tryptic) serve as molecular representatives of the target proteins. In this study, a single tryptic peptide was selected from each of the 142 parent proteins based on peptide selection rules^31–33^, as well as proven peptide detectability in other pooled plasma samples^34, 35^. To help with compensation for matrix-induced suppression or variability in LC-MS performance, C-terminal ^13^C/^15^N-labeled peptide analogues were used as internal standards. These were synthesized (via Fmoc chemistry, with heavy labeled lysine and arginine residues obtained from Cambridge Isotope Laboratories) and purified (through RP-HPLC with subsequent assessment by MALDI-TOF-MS) at the University of Victoria - Genome BC Proteomics Centre, with characterization (via amino acid analysis, AAA, and capillary zone electrophoresis, CZE) conducted at external sites. The CZE-derived purity of the 142 SIS peptides was 94.2%, on average.

### LC/MRM-MS Equipment and Conditions

For details of the solution and sample preparation, the reader is referred to the Supplemental Information section. Fifteen-µL injections of the plasma tryptic digests were separated with a Zorbax Eclipse Plus RP-UHPLC column (2.1 × 150 mm, 1.8 µm particle diameter; on a 1290 Infinity UPLC system (all from Agilent Technologies). Peptide separations were achieved at a flow rate of 0.4 mL∕min over a 43 min run, using a multi-step LC gradient (3–90% mobile phase B; eluent compositions: A was 0.1% FA in 100% H_2_O while B was 0.1% FA in 90% ACN), as described previously^29^. The column and autosampler were maintained at 50 °C and 4 °C, respectively. A post-gradient equilibration time of 4 min was used after the analysis of each sample. All samples were processed individually.

The LC system was interfaced to a triple quadrupole mass spectrometer (Agilent 6490) via Agilent’s Jet Stream™ source, operated in the positive-ion ESI mode. The MRM acquisition parameters employed were identical to those reported previously^29^. Note that the peptide parameters had previously been empirically optimized by direct infusion of the purified SIS peptides. In the quantitative analysis, the targets (852 total transitions for 142 peptides with 3 transitions/peptide form) were monitored during 800-ms cycles and 1-min detection windows.

### Quantitative Analysis

The MRM data was visualized and examined with MassHunter Quantitative Analysis software (version B.07.00; Agilent). This involved peak inspection to ensure accurate selection, integration, and uniformity (in terms of peak shape and retention time) of the SIS and natural (NAT) peptide forms. Standard curves were generated from a pooled control sample with constant NAT and variable levels of SIS peptide concentrations. Qualification of standard curve generation was based on the following criteria:

(i) 1/x^2^ regression weighting with a ‘low-to-high’ concentration removal strategy (defined previously^36^),

(ii) <20% deviation in a level’s precision and accuracy,

(iii) inclusion of all 3 injection replicates from each level, and

(iv) 3 consecutive qualified levels.

Quantitation was done using linear regression analysis, as described previously^29, 36^.

### Statistical Analysis

All of the selected proteins were reliably identified and quantitatively characterized in all plasma samples, with FDR level of <1%. Evaluation of the quality of both the identification and the quantitative analysis was based on the material contained in Appendix 1 (Extended Data File 1). Concentration changes of the identified blood proteins were analyzed by descriptive statistics methods. Analysis of the data revealed its pronounced heterogeneity, as different proteins have different dynamics of concentration changes caused by organism exposure to space flight. To evaluate the level of variability of investigated random variables the ratio of standard deviation to the average mean value was calculated. The following methods of analysis of variance were used to determine significance:  least significant difference (LSD) Test, Scheffe, Newman-Keuls, Tukey honest significant difference (HSD) test, Duncan, Unequal N HSD Tests^37^.

## Results and Discussions

We have previously quantified other large panels of plasma proteins with simple sample preparation and LC/MRM-MS sample processing through a combination of linear regression and single point measurements^29, 31, 34^. Based on these previous studies, a collection of 142 extracellular proteins were selected for interrogation against 54 cosmonaut plasma samples. Preliminary experiments involved concentration-balancing the SIS peptide mixture to the pooled cosmonaut plasma, performing rigorous interference screening through ion ratio analysis, and automating the sample preparation process on a liquid handling robot. Ultimately, 125 plasma proteins were detected and quantitated.

To examine the proteomic changes during space flight, the determined concentrations at 3 time points (labeled L-30, *i.e*., 30 days prior to launch; R + 1, a day after landing and R + 7, 7 days after landing) were assessed independently on a protein-by-protein basis (see Supplemental Information, Table 1). Through this comparison, it was found that the behavior was heterogeneous, as post-flight changes in concentrations were protein-dependent. For example, the mean concentration of IGFALS (insulin-like growth factor binding protein complex, acid labile subunit; UniProtKB: P35858) was relatively stable post-flight, while the levels of cDNA (UniProtKB: B7Z2X4) recovered slowly. For this reason, the proteins were divided into groups (classes) that shared common characteristics.

**Table 1:** P-values for proteins which show significant differences at the p < 0.05 level with the LSD Test.

Protein	Time-points Compared		
	L-30 vs R + 1	L-30 vs R + 7	R + 1 vs R + 7
78 kDa glucose-regulated protein	0.016231-Dec	ns	ns
Alpha-2-antiplasmin	0.032056-Dec	ns	ns
Apolipoprotein A-I	0.005682-Dec	0.029907-Dec	ns
Apolipoprotein A-II	0.049591-Dec	ns	0.003260-Inc
Apolipoprotein C-I	0.048209-Dec	ns	ns
Beta-2-glycoprotein 1	0.028184-Dec	ns	0.002013-Inc
CD5 antigen-like	0.010158-Dec	ns	0.045895-Inc
Complement factor D	0.017977-Dec	ns	ns
Extracellular matrix protein 1	0.022876-Dec	ns	ns
Fetuin-B	ns	0.011128-Dec	0.000363-Inc
Fibrinogen beta chain	0.00032-Dec	ns	0.018719-Inc
Gelsolin	ns	0.016365-Dec	0.041981-Dec
Insulin-like growth factor-binding protein 3	0.017826-Dec	ns	0.002810-Inc
Insulin-like growth factor-binding protein complex acid labile subunit	ns	0.010068-Dec	ns
Kallistatin	ns	ns	0.014792-Inc
Lumican	ns	0.044139-Inc	ns
mRNA for apolipoprotein E	0.012596-Inc	ns	0.043471-Dec
Phospholipid transfer protein	0.016353-Dec	0.025245-Dec	ns
Protein AMBP	ns	0.045234-Dec	ns

Notes to table: L-30–30 days prior to launch; R + 1 – the 1st day after landing; R + 7 – the 7th day after landing; Inc – increased level; Dec – decreased level; ns – no significant difference.

A decrease in the level of certain proteins in the blood, without these levels being restored to their pre-flight levels 7 days after the flight, can clearly be interpreted as the impact of space flight on the human body. Two protein with similar dynamics (apolipoprotein A-II, UniProtKB: P02652; and serotransferrin, UniProtKB: P02787) showed pronounced decreases in variability of their concentrations on the 7^th^ day after landing (Table 1). The second group of proteins is characterized by reduction to below preflight levels, with recovery (or even increases) on the 7^th^ day after the flight. This means that changes in their blood concentrations are transitory and may be attributable to the influence of the final stage of the flight, including factors such as emotional stress and overloads during the landing stage. Finally, the third type of dynamics, identified on the basis of descriptive statistical methods, is characterized by persistence of the initial protein concentration levels until the +1 day after completion of the flight, with an increase/or decrease by the +7^th^ day after landing. We believe that these changes reflect the organism’s rehabilitation to the conditions of the Earth’s gravity, after the period of life in space. Thus, by using a statistical method to compare two sets in this relatively large dataset (125 proteins from 18 cosmonauts with concentrations measured in three time points), it was revealed that the concentrations of only 19 of the 125 proteins were influenced by space flight. In other words, the average concentrations of the majority of proteins did not show statistically significant changes after the flight.

Table 1 shows the 19 proteins with statistically significant differences using the LSD test. Significant differences between time points were confirmed for the same proteins using other methods of analysis–Scheffe, Newman-Keuls, Tukey HSD, Duncan, and Unequal N HSD tests (data not shown).

In order to determine the biological functions of the proteins whose concentrations are changed during the flight, and to identify possible adaptation mechanisms in which they are involved, manual annotation of proteins was carried out using Gene Ontology, PubMed, UniProtKB, REACTOME, KEGG, WikiPathways, and Pathway Interaction. A brief summary of this annotation is given in Appendix 2.

We believe that the proteins whose levels were found to be reduced immediately after the landing of cosmonauts and which had not recovered to pre-flight levels by the +7^th^ day, are responsible for structural adaptation that occurred during flight. The proteins whose concentrations were found at their pre-flight levels after landing, but which underwent changes in the post-flight period, apparently are involved in the rehabilitation process to terrestrial conditions.

In summary, plasma proteins whose concentrations changed during flight included pathways related to oxidative stress, cytoskeleton, cell proliferation, glucose and lipid metabolism, cell damage and repair response, apoptosis, calcium/collagene metabolism, transport of lipoproteins, cellular functions, protein degradation, signal transduction and cell energy metabolism. Mainly non-tissue-specific pathways were affected, since these biological processes are carried out in most human tissues. Moreover, the expression of proteins related to cytoskeleton, extracellular matrix (ECM) metabolism, internal cell transport, cell motility, apoptosis and oxidative stress were altered during the time of exposure to microgravity (μG), suggesting their sensitivity to gravitational changes. Later, after landing, further protein expression changes are probably involved in the adaptation to conditions on earth, suggesting that physiological systems and organism are able to respond to gravitational field changes by increasing the expression of specific defense proteins, thereby reducing any long-term damaging effect of μG.

In this paper, a relatively large-scale study of blood protein concentration changes caused by the conditions of cosmonauts staying on the International Space Station in high-earth orbit has been carried out by quantitative proteomics using targeted, bottom-up UHPLC/MRM-MS analysis. We have found that the plasma concentrations of 85% of the proteins investigated were not significantly altered due to space flight.

Adaptation of all human body functions is carried out with the participation of proteins. Stein, *et al*. stated that the total rate of protein synthesis in the body decreases under the influence of space flight factors^38^. Their data set, obtained during examination of cosmonauts after the completion of the space flight (SF), still remains the most representative. However, other authors who have studied the dynamics of the concentrations of certain proteins in the blood caused by SF, have come to a different conclusion. For example, after the long expedition to the orbital station “Skylab” and short-term missions to the “Shuttle”, no increase in acute phase proteins was found^39^, but a survey of 29 astronauts after long-term space missions (from 125 to 366 days) showed that on the 2nd day after landing, the relative content of α-1 and α-2 globulin fractions increased, which is a sign of “acute” phase reaction^40^. There is also a discrepancy in the quantitative results for immunoglobulins G, A, and M concentrations after long expeditions: Guseva and Tashpulatov [1980] found them to be increased^41^, while Rykova, *et al*. [2001] did not find significant changes in 53 cosmonauts^42^. In a study of the biochemical status of astronauts after long-term (78–208 days, 52 people) and extra-long-term space missions (240–438 days, 7 persons) on the orbital station “Mir”, significant changes in albumin and α-1-, α-2-, β-, and γ-globulin were found, and ﻿the total plasma protein concentration was slightly but significantly decreased^43^. Leach, *et al*. [1983] reported that albumin concentration decreased during space flight by 50% or more compared with baseline values^44^. Thus, the data obtained by different investigators often does not agree with each other. This circumstance may have several causes: 1) data were obtained using methods with different analytical specificity and sensitivity. 2) In different experiments, the sets of proteins studied were different and the data were obtained on different biological subjects (rats, mice, monkeys, and humans)^45^. For these reasons, it is not possible to combine all these data into ﻿a single set which could be used to construct a picture of specific SF﻿-induced﻿ changes, either on the pathway level or on the physiological level. In other words, we still cannot﻿ be certain which proteins are associated with which physiological effects. We believe that the metabolic shifts recorded after extended space missions are caused by a broad range of biochemical processes. The most pronounced of these are the decrease in intensity of biological oxidation^46, 47^; inhibition of the rate of protein synthesis and heat generation^48^; activation of gluconeogenesis^43, 44^; oxidative stress^38, 48^; reduction of the iron store capacity in organism^49^; lipolysis activation^43, 44^; hypohydration of organism^5^; and inhibition of natural and adaptive immunity^25, 50, 51^.

In the review by Grigoriev *et al*.^52^, based on a generalization of the available biochemical and morphological evidence, it was hypothesized that a decrease in all physiological processes occurred during space travel. In 2003, Biolo, *et al*., in their paper entitled,  “Microgravity as a model of ageing”, hypothesized that most of the changes induced by space flight were identical to those that occur during the process of aging on Earth^53^.

Before the appearance of post-genome era technologies, it was not possible to get the reliable picture of physiological adaptive changes induced in human organism during extended space mission. Current methods of quantitative proteomics provide us with the opportunity of achieving this goal.

Contrary to expectation, statistically significant reductions in the plasma concentrations found for only a small number of the 125 proteins quantified in the 18 cosmonauts (Supplemental Information, Table 1). Analysis of the biological processes in which these proteins are involved showed that virtually all of these plasma proteins are linked to a network of mechanisms that regulate protease activities, natural immunity, lipid turnover, coagulation pathways, and metabolism of the extracellular matrix. MacLean has hypothesized that similar effects on extracellular signaling pathways could be triggered by direct and indirect mechanotransduction^54^. A mutual coordination of proteins participating in regulation of the complement, lipid turnover, acute stage proteins, and proteinase inhibitors has also been observed by many authors^55, 56^. The complement system in mammals contributes to regulation of different functions including homeostasis maintenance, and support of liver, bone, and muscle regeneration^57^. In our study, Neurofilin-2 (NRP2; UniProtKB: O60462), is a protein whose plasma concentration first decreases and then returns to its original concentration within seven days after space flight. The post-flight behavior of this and several other proteins that were first observed in our study supports hypotheses concerning these protein-protein interactions, and their verification using the system phenomena recognized in gravitational physiology.

Consequently, our results show the adaptive changes in the extracellular concentration of proteins induced by spaceflight. The picture is different from that obtained from studies in the field of environmental medicine and gerontology – for example, in contrast to aging, adaptation to the space flight is a reversible process, although the recovery of certain tissues is very slow. Thus, valuable knowledge about the biological diversity of the processes associated with dynamically synthesized proteins has been obtained from our study. Our results support the hypothesis that adaptation to the conditions of space flight takes place in all of the major types of human cells, tissues, and organs. Although our research was carried out on a relatively large set of target proteins, it could not cover the entire diversity of proteins within a biological sample. This would require an expanded set of target proteins. The selection of these target proteins could be made on the basis of metabolomics data, using pathway analysis.

Weightlessness for human is completely new in evolutionary terms, being an environmental factor our species has not faced during the course of evolution. Therefore the adaptation mechanisms of the human organism to these conditions is not predictable, nor is the set of protein that is affected by these adaptation processes. In this study, we quantified a set of proteins that carry out their function in the extracellular fluid, and which are used in the clinic for diagnosing non-communicable diseases (e.g., metabolic syndrome)^29^. To select proteins specifically involved in adaptation to weightlessness, the most appropriate way would be to predict them from gene-based pathways which are associated with the reactions leading to the well-known physiological effects of microgravity. Currently, however, the accuracy of the approaches based on genomic pathways is not sufficient to predict the involvement of particular genes in microgravity-induced changes. Thus, by conducting research on the proteome level and selecting proteins that are significantly modulated by weightlessness, we could get a new list of proteins involved in this particular adaptation. These, in turn, will be targeted during the subsequent stages of this work.

## Electronic supplementary material

Dataset 1
