# Publisher Correction: Protein expression changes caused by spaceflight as measured for 18 Russian cosmonauts

**DOI:** 10.1038/s41598-019-43121-w

**Published:** 2019-06-07

**Authors:** Irina M. Larina, Andrew J. Percy, Juncong Yang, Christoph H. Borchers, Andrei M. Nosovsky, Anatoli I. Grigoriev, Evgeny N. Nikolaev

**Affiliations:** 1 Institute of Biomedical Problems, Russian Academy of Sciences, 76a Khoroshevskoye shosse, 123007 Moscow, Russia; 2 University of Victoria - Genome BC Proteomics Centre, University of Victoria, Vancouver Island Technology Park, 3101–4464 Markham Street, Victoria, British Columbia V8Z 7X8 Canada; 3 Department of Biochemistry and Microbiology, University of Victoria, Petch Building Room 207, 3800 Finnerty Road, Victoria, British Columbia V8P 5C2 Canada; 4 Lady Davis Institute, Jewish General Hospital, McGill University, 3755 Côte Ste-Catherine Road, Montreal, Quebec, H3T 1E2 Canada; 5 Gerald Bronfman Department of Oncology, Jewish General Hospital, 3755 Côte Ste-Catherine Road, Montreal, Quebec, H3T 1E2 Canada; 6 Skolkovo Institute of Science and Technology, Skoltech, Moscow region, Skolkovo village, Novaya st.100, 143025 Russia; 7 Moscow Institute of Physics and Technology, Moscow region, Dolgoprudnyj, Institutskij pr. 9, 141700 Russia; 8 Institute of Energy Problems of Chemical Physics Russian Academy of Sciences, 38 k.2 Leninskij pr., 119334 Moscow, Russia

Correction to: *Scientific Reports* 10.1038/s41598-017-08432-w, published online 15 August 2017

In the original version of this Article, the authors Irina M. Larina, Andrei M. Nosovsky, Anatoli I. Grigoriev and Evgeny N. Nikolaev were incorrectly indexed. This error has now been corrected.

