# Supplementary material for: Protein expression changes caused by spaceflight as measured for 18 Russian cosmonauts
Source: Sci Rep. 2017 Aug 15;7:8142. doi: 10.1038/s41598-017-08432-w (PMC5557884; doi:10.1038/s41598-017-08432-w)
Supplement: Supplementary file 1 — Dataset 1 [file 41598_2017_8432_MOESM1_ESM.doc]

**Supplementary Information**

**Protein expression changes caused by spaceflight as measured for 18 Russian cosmonauts**

Irina Larina^1^, Andrew J. Percy^2^, Juncong Yang^2^, Christoph H. Borchers^2-5^, Andrei Nosovsky^1^, Anatoli Grigoriev^1^, and Evgeny Nikolaev^6-8*^

^1^Institute of Biomedical Problems, Russian Academy of Sciences, 76a Khoroshevskoye shosse, 123007, Moscow, Russia

^2^University of Victoria - Genome BC Proteomics Centre, University of Victoria, Vancouver Island Technology Park, Victoria, British Columbia V8Z 7X8, Canada

^3^Department of Biochemistry and Microbiology, University of Victoria, Petch Building Room 207, Victoria, British Columbia V8P 5C2, Canada

^4^Lady Davis Institute, Jewish General Hospital, 3755 Côte Ste-Catherine Road, McGill University, Montreal, Quebec, H3T 1E2, Canada

5Gerald Bronfman Department of Oncology, Jewish General Hospital, 3755 Côte Ste-Catherine Road, .Montreal, Quebec, H3T 1E2, Canada

^6^Skolkovo Institute of Science and Technology, Skoltech, Moscow region, Skolkovo village, Novaya st.100, 143025, Russia

^7^Moscow Institute of Physics and Technology, Moscow region, Dolgoprudnyj. Institutskij pr. 9, 141700, Russia

^8^Institute of Energy Problems of Chemical Physics Russian Academy of Sciences, 38 k.2 Leninskij pr.,119334, Moscow, Russia

**Table of Contents**

**Page**

**Supplemental Information – Methods** S3

**Supplemental Information Table 1** S4

**Supplemental Information -- Methods**

*Solution and Sample Preparations*

The plasma proteolytic digests were prepared in an automated manner on a Tecan Freedom EVO 150 workstation. All of the liquid handling steps associated with plasma protein preparation were automated (including denaturation, disulfide bond reduction, and cysteine alkylation, tryptic digestion, SIS peptide addition, and solid phase extraction (SPE)) on a 96-well microplate (Axygen). For sample preparation, 30 µL of 1/10 plasma (8 µL raw fluid consumed per sample) was subjected to 10% sodium deoxycholate, 50 mM TCEP, 100 mM iodoacetamide, and 100 mM dithiothreitol (all in 25 mM ammonium bicarbonate). Denaturation and reduction proceeded simultaneously for 30 min at 60°C, with alkylation and quenching occurring subsequently for 30 min at 37°C. Proteolysis was initiated by the addition of TPCK-treated trypsin (10.5 µL at 2 mg/mL; Worthington) at a 10:1 substrate:enzyme ratio. After overnight incubation at 37ºC, proteolysis was terminated and the acid-insoluble surfactant was pelleted by the addition of a chilled acidified SIS peptide mixture (30 µL of 250-2.5 fmol/µL for the pooled control or 25 fmol/µL for the individual samples), followed by the addition of 1.9% FA to each digest aliquot. Note that the control consisted of pooled aliquots of the 18 pre-flight samples (30 days prior to launch, L-30). The SIS concentrations spanned a 100-fold concentration range (dilution series: 1:2:5:1.67:3:2, from most to least concentrated) to generate the standard curves for the control sample, with a constant amount of concentration-balanced SIS peptide mixture spiked into each subject’s sample. Following acidification, the peptide-containing supernatant was concentrated by solid phase extraction (Waters Oasis HLB, 2 mg sorbent), and then lyophilized to dryness and rehydrated in 0.1% FA (final concentration: 1 µg/µL digest) for LC/MRM-MS.

**Supplemental Information Table 1: Protein concentrations in cosmonaut plasma: changes with respect to long-duration spaceflight (SF).**

| **Protein Name** | **UniProtKB Accesssion number** | **Gene Name** | **Peptide** | **n** | **Time Points** | **Mean (fmol/µL)** | **Std. Dev.** |
| --- | --- | --- | --- | --- | --- | --- | --- |
| 78 kDa glucose-regulated protein | P11021 | HSPA5 | ITPSYVAFTPEGER | 9 | Before SF | 6.27 | 1.86 |
|  |  |  |  | 10 | R+1 * | 4.6 | 1.15 |
|  |  |  |  | 8 | R+7 | 5.28 | 0.99 |
| Actin, alpha cardiac muscle 1 | P68032 | ACTC1 | DSYVGDEAQSK | 14 | Before SF | 21.60 | 33.18 |
|  |  |  |  | 16 | R+1 | 9.83 | 6.83 |
|  |  |  |  | 18 | R+7 | 8.58 | 4.90 |
| Afamin | P43652 | AFM | IAPQLSTEELVSLGEK | 18 | Before SF | 1003.95 | 224.89 |
|  |  |  |  | 18 | R+1 | 905.53 | 171.11 |
|  |  |  |  | 18 | R+7 | 946.07 | 194.21 |
| Alpha-1-acid glycoprotein 1 | P02763 | ORM1 | NWGLSVYADKPETTK | 8 | Before SF | 8.31 | 7.61 |
|  |  |  |  | 11 | R+1 | 5.38 | 2.04 |
|  |  |  |  | 11 | R+7 | 5.18 | 2.15 |
| Alpha-1-antichymotrypsin | P01011 | SERPINA3 | EIGELYLPK | 18 | Before SF | 2392.50 | 785.41 |
|  |  |  |  | 18 | R+1 | 2411.61 | 707.15 |
|  |  |  |  | 18 | R+7 | 2473.10 | 555.81 |
| Alpha-1-antitrypsin | P01009 | SERPINA1 | LSITGTYDLK | 18 | Before SF | 29683.32 | 5392.80 |
|  |  |  |  | 18 | R+1 | 28944.95 | 5027.14 |
|  |  |  |  | 18 | R+7 | 29385.33 | 3382.42 |
| Alpha-1B-glycoprotein | P04217 | A1BG | LETPDFQLFK | 18 | Before SF | 2630.23 | 415.95 |
|  |  |  |  | 18 | R+1 | 2595.97 | 450.77 |
|  |  |  |  | 18 | R+7 | 2829.48 | 389.63 |
| Alpha-2-antiplasmin | P08697 | SERPINF2 | LGNQEPGGQTALK | 18 | Before SF | 489.39 | 106.12 |
|  |  |  |  | 18 | R+1 | 446.95 | 100.04 |
|  |  |  |  | 18 | R+7 | 450.72 | 87.34 |
| Alpha-2-HS-glycoprotein | P02765 | AHSG | APHGPGLIYR | 13 | Before SF | 134.15 | 75.89 |
|  |  |  |  | 12 | R+1 * | 80.32 | 38.13 |
|  |  |  |  | 5 | R+7 | 86.26 | 51.08 |
| Alpha-2-macroglobulin | P01023 | A2M | TEHPFTVEEFVLPK | 18 | Before SF | 1843.21 | 999.98 |
|  |  |  |  | 18 | R+1 | 1461.20 | 364.46 |
|  |  |  |  | 18 | R+7 | 1502.76 | 315.05 |
| Angiotensinogen | P01019 | AGT | ALQDQLVLVAAK | 18 | Before SF | 121.08 | 64.47 |
|  |  |  |  | 18 | R+1 | 112.36 | 35.08 |
|  |  |  |  | 18 | R+7 | 113.31 | 20.08 |
| Antithrombin-III | P01008 | SERPINC1 | TSDQIHFFFAK | 18 | Before SF | 570.55 | 169.74 |
|  |  |  |  | 18 | R+1 | 551.23 | 140.19 |
|  |  |  |  | 18 | R+7 | 559.15 | 122.09 |
| Apolipoprotein A-I | P02647 | APOA1 | ATEHLSTLSEK | 18 | Before SF | 39127.13 | 6925.42 |
|  |  |  |  | 18 | R+1 | 36462.78 | 5826.00 |
|  |  |  |  | 18 | R+7 | 40033.76 | 7611.30 |
| Apolipoprotein A-II | P02652 | APOA2 | SPELQAEAK | 18 | Before SF | 5168.84 | 1327.41 |
|  |  |  |  | 18 | R+1 * | 4188.92 | 940.56 |
|  |  |  |  | 18 | R+7 * | 4410.78 | 680.19 |
| Apolipoprotein A-IV | P06727 | APOA4 | LLPHANEVSQK | 18 | Before SF | 1277.67 | 438.36 |
|  |  |  |  | 18 | R+1 * | 1002.14 | 434.20 |
|  |  |  |  | 18 | R+7 ** | 1425.11 | 354.96 |
| Apolipoprotein B-100 | P04114 | APOB | FPEVDVLTK | 18 | Before SF | 474.89 | 107.23 |
|  |  |  |  | 18 | R+1 | 478.05 | 144.94 |
|  |  |  |  | 18 | R+7 | 415.89 | 95.76 |
| Apolipoprotein C-I | P02654 | APOC1 | TPDVSSALDK | 18 | Before SF | 41.68 | 35.44 |
|  |  |  |  | 18 | R+1 | 30.22 | 8.79 |
|  |  |  |  | 18 | R+7 | 34.91 | 9.79 |
| Apolipoprotein C-II | P02655 | APOC2 | ESLSSYWESAK | 18 | Before SF | 440.28 | 326.31 |
|  |  |  |  | 18 | R+1 * | 289.39 | 144.89 |
|  |  |  |  | 18 | R+7 | 361.57 | 150.22 |
| Apolipoprotein C-III | P02656 | APOC3 | GWVTDGFSSLK | 17 | Before SF | 8.11 | 3.03 |
|  |  |  |  | 17 | R+1 | 6.70 | 2.01 |
|  |  |  |  | 18 | R+7 | 8.27 | 2.11 |
| Apolipoprotein C-IV | P55056 | APOC4 | GFMQTYYDDHLR | 15 | Before SF | 18.08 | 10.32 |
|  |  |  |  | 17 | R+1 | 14.01 | 9.68 |
|  |  |  |  | 16 | R+7 | 15.43 | 6.58 |
| Apolipoprotein D | P05090 | APOD | VLNQELR | 18 | Before SF | 2950.53 | 734.06 |
|  |  |  |  | 18 | R+1 | 2612.49 | 801.78 |
|  |  |  |  | 18 | R+7 | 2921.19 | 738.90 |
| Apolipoprotein E | P02649 | APOE | LGPLVEQGR | 18 | Before SF | 797.87 | 196.76 |
|  |  |  |  | 18 | R+1 | 914.50 | 256.19 |
|  |  |  |  | 18 | R+7 | 915.79 | 226.36 |
| Apolipoprotein F | Q13790 | APOF | SGVQQLIQYYQDQK | 18 | Before SF | 34.65 | 12.19 |
|  |  |  |  | 18 | R+1 | 31.74 | 7.84 |
|  |  |  |  | 18 | R+7 | 29.73 | 5.93 |
| Apolipoprotein L1 | O14791 | APOL1 | VTEPISAESGEQVER | 18 | Before SF | 260.37 | 93.53 |
|  |  |  |  | 18 | R+1 | 243.44 | 71.47 |
|  |  |  |  | 18 | R+7 | 250.39 | 89.23 |
| Apolipoprotein M | O95445 | APOM | AFLLTPR | 18 | Before SF | 2018.17 | 366.10 |
|  |  |  |  | 18 | R+1 | 1963.01 | 373.43 |
|  |  |  |  | 18 | R+7 | 2061.98 | 377.82 |
| Apolipoprotein(a) | P08519 | LPA | GTYSTTVTGR | 18 | Before SF | 341.51 | 363.61 |
|  |  |  |  | 18 | R+1 | 295.13 | 331.46 |
|  |  |  |  | 18 | R+7 | 240.94 | 324.12 |
| Beta-2-glycoprotein 1 | P02749 | APOH | ATVVYQGER | 18 | Before SF | 3367.24 | 564.90 |
|  |  |  |  | 18 | R+1 | 3387.25 | 863.15 |
|  |  |  |  | 18 | R+7 | 3471.08 | 560.60 |
| Beta-2-microglobulin | P61769 | B2M | VNHVTLSQPK | 18 | Before SF | 110.62 | 16.59 |
|  |  |  |  | 18 | R+1 * | 96.56 | 18.98 |
|  |  |  |  | 18 | R+7 ** | 116.82 | 20.25 |
| Beta-Ala-His dipeptidase | Q96KN2 | CNDP1 | ALEQDLPVNIK | 18 | Before SF | 35.43 | 20.44 |
|  |  |  |  | 18 | R+1 | 33.34 | 10.58 |
|  |  |  |  | 18 | R+7 | 37.26 | 9.61 |
| C4b-binding protein alpha chain | P04003 | C4BPA | LNNGEITQHR | 18 | Before SF | 208.06 | 60.39 |
|  |  |  |  | 18 | R+1 | 208.94 | 49.78 |
|  |  |  |  | 18 | R+7 | 211.13 | 53.63 |
| Cadherin-5 | P33151 | CDH5 | YEIVVEAR | 18 | Before SF | 16.23 | 3.04 |
|  |  |  |  | 18 | R+1 | 14.19 | 2.82 |
|  |  |  |  | 18 | R+7 | 15.68 | 3.83 |
| Carbonic anhydrase 1 | P00915 | CA1 | VLDALQAIK | 18 | Before SF | 55.45 | 56.40 |
|  |  |  |  | 18 | R+1 | 69.91 | 99.70 |
|  |  |  |  | 18 | R+7 | 56.78 | 43.95 |
| Carboxypeptidase B2 | Q96IY4 | CPB2 | DHEELSLVASEAVR | 17 | Before SF | 12.26 | 3.48 |
|  |  |  |  | 18 | R+1 | 12.18 | 2.77 |
|  |  |  |  | 18 | R+7 | 13.03 | 2.97 |
| Cathelicidin antimicrobial peptide | P49913 | CAMP | AIDGINQR | 18 | Before SF | 15.48 | 6.18 |
|  |  |  |  | 18 | R+1 | 13.81 | 3.96 |
|  |  |  |  | 18 | R+7 | 16.88 | 6.12 |
| CD44 antigen | P16070 | CD44 | YGFIEGHVVIPR | 18 | Before SF | 33.80 | 6.74 |
|  |  |  |  | 18 | R+1 | 30.78 | 6.05 |
|  |  |  |  | 18 | R+7 | 34.29 | 6.43 |
| CD5 antigen-like | O43866 | CD5L | LVGGLHR | 18 | Before SF | 1240.15 | 356.19 |
|  |  |  |  | 18 | R+1 | 1113.42 | 327.98 |
|  |  |  |  | 18 | R+7 | 1126.31 | 392.90 |
| cDNA FLJ53327 | B7Z2X4 |  | EGGQTAPASTR | 18 | Before SF | 1007.09 | 231.19 |
|  |  |  |  | 18 | R+1 * | 802.18 | 232.81 |
|  |  |  |  | 18 | R+7 ** | 959.24 | 226.75 |
| Ceruloplasmin | P00450 | CP | GAYPLSIEPIGVR | 18 | Before SF | 2158.26 | 632.13 |
|  |  |  |  | 18 | R+1 | 2182.89 | 439.44 |
|  |  |  |  | 18 | R+7 | 2292.21 | 348.84 |
| Cholinesterase | P06276 | BCHE | YLTLNTESTR | 18 | Before SF | 30.45 | 9.84 |
|  |  |  |  | 18 | R+1 | 27.51 | 6.82 |
|  |  |  |  | 18 | R+7 | 25.62 | 7.96 |
| Clusterin | P10909 | CLU | ELDESLQVAER | 18 | Before SF | 2861.57 | 438.51 |
|  |  |  |  | 18 | R+1 | 2824.34 | 507.37 |
|  |  |  |  | 18 | R+7 | 2942.84 | 485.07 |
| Coagulation factor IX | P00740 | F9 | SALVLQYLR | 18 | Before SF | 11.02 | 5.48 |
|  |  |  |  | 18 | R+1 | 10.07 | 2.82 |
|  |  |  |  | 18 | R+7 | 11.66 | 2.77 |
| Coagulation factor VII | P08709 | F5 | VAQVIIPSTYVPGTTNHDIALLR | 18 | Before SF | 7.23 | 1.24 |
|  |  |  |  | 18 | R+1 | 7.13 | 1.30 |
|  |  |  |  | 18 | R+7 | 7.67 | 1.33 |
| Coagulation factor X | P00742 | F10 | TGIVSGFGR | 18 | Before SF | 155.76 | 34.17 |
|  |  |  |  | 18 | R+1 | 147.08 | 27.08 |
|  |  |  |  | 18 | R+7 | 166.90 | 32.09 |
| Coagulation factor XI | P03951 | F11 | TSESGLPSTR | 18 | Before SF | 46.91 | 14.66 |
|  |  |  |  | 18 | R+1 | 49.30 | 13.99 |
|  |  |  |  | 18 | R+7 | 46.24 | 10.69 |
| Coagulation factor XII | P00748 | F12 | TEQAAVAR | 18 | Before SF | 494.25 | 162.01 |
|  |  |  |  | 18 | R+1 | 437.20 | 120.42 |
|  |  |  |  | 18 | R+7 | 473.00 | 124.45 |
| Complement C1q subcomponent subunit A | P02745 | C1QA | SLGFCDTTNK | 18 | Before SF | 11.04 | 5.79 |
|  |  |  |  | 18 | R+1 | 9.35 | 2.67 |
|  |  |  |  | 18 | R+7 | 10.71 | 2.97 |
| Complement C1q subcomponent subunit B | P02746 | C1QB | IAFSATR | 18 | Before SF | 416.79 | 71.40 |
|  |  |  |  | 18 | R+1 | 373.78 | 66.93 |
|  |  |  |  | 18 | R+7 | 385.43 | 62.07 |
| Complement C1q subcomponent subunit C | P02747 | C1QC | FQSVFTVTR | 18 | Before SF | 695.84 | 137.14 |
|  |  |  |  | 18 | R+1 | 636.48 | 134.12 |
|  |  |  |  | 18 | R+7 | 693.14 | 131.04 |
| Complement C1r subcomponent | P00736 | C1R | GLTLHLK | 18 | Before SF | 376.43 | 71.97 |
|  |  |  |  | 18 | R+1 | 363.55 | 74.25 |
|  |  |  |  | 18 | R+7 | 404.66 | 89.00 |
| Complement C1s subcomponent | P09871 | C1S | SDFSNEER | 18 | Before SF | 250.73 | 114.46 |
|  |  |  |  | 18 | R+1 | 211.63 | 49.57 |
|  |  |  |  | 18 | R+7 | 233.83 | 51.65 |
| Complement C2 | P06681 | C2 | HAFILQDTK | 18 | Before SF | 108.41 | 22.01 |
|  |  |  |  | 18 | R+1 | 109.51 | 24.65 |
|  |  |  |  | 18 | R+7 | 110.01 | 20.90 |
| Complement C3 | P01024 | C3 | SGIPIVTSPYQIHFTK | 18 | Before SF | 5936.38 | 1221.20 |
|  |  |  |  | 18 | R+1 | 5986.70 | 1209.81 |
|  |  |  |  | 18 | R+7 | 6141.22 | 976.44 |
| Complement C4-B | P0C0L5 | C4B | VGDTLNLNLR | 18 | Before SF | 1639.52 | 353.25 |
|  |  |  |  | 18 | R+1 | 1651.66 | 301.09 |
|  |  |  |  | 18 | R+7 | 1691.31 | 349.93 |
| Complement C5 | P01031 | C5 | IDTQDIEASHYR | 18 | Before SF | 89.10 | 39.49 |
|  |  |  |  | 18 | R+1 | 79.64 | 18.49 |
|  |  |  |  | 18 | R+7 | 79.88 | 16.95 |
| Complement component 4 | P0C0L5 | C4B | DHAVDLIQK | 18 | Before SF | 801.26 | 370.35 |
|  |  |  |  | 18 | R+1 | 713.14 | 220.24 |
|  |  |  |  | 18 | R+7 | 787.37 | 246.65 |
| Complement component C7 | P10643 | C7 | LIDQYGTHYLQSGSLGGEYR | 18 | Before SF | 501.95 | 144.59 |
|  |  |  |  | 18 | R+1 | 483.46 | 121.45 |
|  |  |  |  | 18 | R+7 | 512.55 | 129.24 |
| Complement component C8 alpha chain | P07357 | C8A | MESLGITSR | 18 | Before SF | 84.99 | 29.71 |
|  |  |  |  | 18 | R+1 | 81.48 | 23.03 |
|  |  |  |  | 18 | R+7 | 82.52 | 17.34 |
| Complement component C9 | P02748 | C9 | VVEESELAR | 18 | Before SF | 215.00 | 57.42 |
|  |  |  |  | 18 | R+1 | 252.29 | 60.48 |
|  |  |  |  | 18 | R+7 | 247.21 | 63.75 |
| Complement factor B | P00751 | CFB | EELLPAQDIK | 18 | Before SF | 157.51 | 61.64 |
|  |  |  |  | 18 | R+1 | 143.57 | 40.98 |
|  |  |  |  | 18 | R+7 | 156.66 | 38.26 |
| Complement factor D | P00746 | CFD | THHDGAITER | 18 | Before SF | 40.85 | 5.34 |
|  |  |  |  | 18 | R+1 | 36.93 | 7.20 |
|  |  |  |  | 18 | R+7 | 38.57 | 6.99 |
| Complement factor H | P08603 | CFH | IDVHLVPDR | 18 | Before SF | 159.24 | 66.85 |
|  |  |  |  | 18 | R+1 | 138.22 | 38.92 |
|  |  |  |  | 18 | R+7 | 150.37 | 35.17 |
| Complement factor I | P05156 | CFI | VFSLQWGEVK | 18 | Before SF | 358.39 | 102.46 |
|  |  |  |  | 18 | R+1 | 342.14 | 88.21 |
|  |  |  |  | 18 | R+7 | 388.73 | 90.52 |
| Cystatin-C | P01034 | CST3 | ASNDMYHSR | 13 | Before SF | 71.94 | 32.60 |
|  |  |  |  | 12 | R+1 * | 41.87 | 22.52 |
|  |  |  |  | 5 | R+7 | 49.04 | 37.62 |
| Endothelial protein C receptor | Q9UNN8 | PROCR | TLAFPLTIR | 18 | Before SF | 6.24 | 3.24 |
|  |  |  |  | 18 | R+1 | 5.19 | 1.98 |
|  |  |  |  | 18 | R+7 | 6.33 | 3.06 |
| Extracellular matrix protein 1 | Q16610 | ECM1 | ELPSLQHPNEQK | 17 | Before SF | 14.52 | 6.61 |
|  |  |  |  | 16 | R+1 | 13.03 | 7.05 |
|  |  |  |  | 15 | R+7 | 14.65 | 6.28 |
| Fetuin-B | Q9UGM5 | FETUB | LVVLPFPK | 18 | Before SF | 46.65 | 16.15 |
|  |  |  |  | 18 | R+1 | 45.17 | 15.91 |
|  |  |  |  | 18 | R+7 | 50.23 | 16.12 |
| Fibrinogen alpha chain | P02671 | FGA | GSESGIFTNTK | 18 | Before SF | 6781.88 | 2899.34 |
|  |  |  |  | 18 | R+1 | 7651.44 | 1349.96 |
|  |  |  |  | 18 | R+7 | 7726.83 | 1492.38 |
| Fibrinogen beta chain | P02675 | FGB) | HQLYIDETVNSNIPTNLR | 16 | Before SF | 418.97 | 93.18 |
|  |  |  |  | 18 | R+1 | 423.54 | 82.00 |
|  |  |  |  | 18 | R+7 | 428.68 | 74.40 |
| Fibrinogen gamma chain | P02679 | FGG | YEASILTHDSSIR | 18 | Before SF | 426.37 | 213.59 |
|  |  |  |  | 18 | R+1 | 480.27 | 146.87 |
|  |  |  |  | 18 | R+7 | 490.05 | 126.52 |
| Fibrinopeptide A | P02671 | FGA | ADSGEGDFLAEGGGVR | 18 | Before SF | 1468.98 | 600.63 |
|  |  |  |  | 18 | R+1 | 1593.31 | 314.40 |
|  |  |  |  | 18 | R+7 | 1663.87 | 303.62 |
| Fibronectin | P02751 | FN1 | NTFAEVTGLSPGVTYYFK | 18 | Before SF | 121.77 | 36.48 |
|  |  |  |  | 18 | R+1 * | 100.85 | 21.73 |
|  |  |  |  | 18 | R+7 | 110.90 | 18.51 |
| Fibulin-1 | P23142 | FBLN1 | TGYYFDGISR | 18 | Before SF | 139.92 | 29.03 |
|  |  |  |  | 18 | R+1 | 129.15 | 21.28 |
|  |  |  |  | 18 | R+7 * ** | 163.82 | 30.46 |
| Galectin-3-binding protein | Q08380 | LGALS3BP | ELSEALGQIFDSQR | 18 | Before SF | 52.81 | 21.06 |
|  |  |  |  | 18 | R+1 | 48.05 | 21.27 |
|  |  |  |  | 18 | R+7 | 53.94 | 18.02 |
| Gelsolin | P06396 | GSN | AGALNSNDAFVLK | 18 | Before SF | 811.56 | 202.65 |
|  |  |  |  | 18 | R+1 * | 611.09 | 138.26 |
|  |  |  |  | 18 | R+7 ** | 737.20 | 112.29 |
| Haptoglobin | P00738 | HP | VGYVSGWGR | 18 | Before SF | 18033.44 | 13376.20 |
|  |  |  |  | 18 | R+1 | 19230.77 | 11329.39 |
|  |  |  |  | 18 | R+7 | 19612.70 | 11127.74 |
| Hemoglobin subunit alpha | P69905 | HBA1 | VGAHAGEYGAEALER | 18 | Before SF | 2628.54 | 2583.05 |
|  |  |  |  | 18 | R+1 | 3248.13 | 5803.66 |
|  |  |  |  | 18 | R+7 | 2452.49 | 2171.83 |
| Hemopexin | P02790 | HPX | NFPSPVDAAFR | 18 | Before SF | 9661.76 | 2388.63 |
|  |  |  |  | 18 | R+1 | 8560.06 | 1776.83 |
|  |  |  |  | 18 | R+7 | 8908.98 | 1696.36 |
| Heparin cofactor 2 | P05546 | SERPIND1 | TLEAQLTPR | 18 | Before SF | 982.29 | 290.18 |
|  |  |  |  | 18 | R+1 | 924.34 | 280.91 |
|  |  |  |  | 18 | R+7 | 1027.53 | 218.13 |
| Histidine-rich glycoprotein | P04196 | HRG | ADLFYDVEALDLESPK | 18 | Before SF | 13120.14 | 1523.55 |
|  |  |  |  | 18 | R+1 | 12418.97 | 1856.16 |
|  |  |  |  | 18 | R+7 | 12679.32 | 1632.85 |
| Hyaluronan-binding protein 2 | Q14520 | HABP2 | VVLGDQDLK | 18 | Before SF | 13.92 | 10.67 |
|  |  |  |  | 18 | R+1 | 10.84 | 2.54 |
|  |  |  |  | 18 | R+7 | 11.51 | 2.45 |
| Insulin-like growth factor binding protein, acid labile subunit | Q8TAY0 | IGFALS | VAGLLEDTFPGLLGLR | 18 | Before SF | 155.60 | 15.44 |
|  |  |  |  | 18 | R+1 | 153.51 | 17.14 |
|  |  |  |  | 18 | R+7 * ** | 142.50 | 14.81 |
| Insulin-like growth factor-binding protein 3 | P17936 | IGFBP3 | FLNVLSPR | 18 | Before SF | 60.82 | 15.05 |
|  |  |  |  | 18 | R+1 | 59.70 | 13.25 |
|  |  |  |  | 18 | R+7 | 57.01 | 14.76 |
| Insulin-like growth factor-binding protein complex acid labile subunit | P35858 | IGFALS | LAELPADALGPLQR | 18 | Before SF | 143.27 | 31.10 |
|  |  |  |  | 18 | R+1 | 156.91 | 30.22 |
|  |  |  |  | 18 | R+7 | 148.14 | 31.60 |
| Inter-alpha-trypsin inhibitor heavy chain H1 | P19827 | ITIH1 | GSLVQASEANLQAAQDFVR | 18 | Before SF | 402.79 | 132.44 |
|  |  |  |  | 18 | R+1 | 362.77 | 62.21 |
|  |  |  |  | 18 | R+7 | 360.16 | 50.01 |
| Inter-alpha-trypsin inhibitor heavy chain H2 | P19823 | ITIH2 | FYNQVSTPLLR | 18 | Before SF | 1656.67 | 318.55 |
|  |  |  |  | 18 | R+1 | 1589.39 | 309.21 |
|  |  |  |  | 18 | R+7 | 1596.91 | 274.80 |
| Intercellular adhesion molecule 1 | P05362 | ICAM1 | LLGIETPLPK | 14 | Before SF | 2.35 | 0.47 |
|  |  |  |  | 9 | R+1 | 2.01 | 0.45 |
|  |  |  |  | 13 | R+7 | 2.40 | 0.47 |
| Kallistatin | P29622 | SERPINA4 | VGSALFLSHNLK | 18 | Before SF | 13.07 | 14.29 |
|  |  |  |  | 18 | R+1 | 8.37 | 2.74 |
|  |  |  |  | 18 | R+7 | 9.25 | 2.28 |
| Kininogen-1 | P01042 | KNG1 | DIPTNSPELEETLTHTITK | 18 | Before SF | 6077.82 | 1566.76 |
|  |  |  |  | 18 | R+1 | 5359.94 | 930.64 |
|  |  |  |  | 18 | R+7 | 5772.22 | 787.41 |
| Leucine-rich alpha-2-glycoprotein | P02750 | LRG1 | ALGHLDLSGNR | 18 | Before SF | 271.23 | 126.00 |
|  |  |  |  | 18 | R+1 | 321.17 | 117.98 |
|  |  |  |  | 18 | R+7 | 308.20 | 55.07 |
| Lipopolysaccharide-binding protein | P18428 | LBP | GLQYAAQEGLLALQSELLR | 14 | Before SF | 121.09 | 31.92 |
|  |  |  |  | 15 | R+1 | 126.79 | 34.84 |
|  |  |  |  | 16 | R+7 | 119.42 | 21.54 |
| L-selectin | P14151 | SELL | AEIEYLEK | 18 | Before SF | 21.67 | 7.43 |
|  |  |  |  | 18 | R+1 | 18.98 | 4.32 |
|  |  |  |  | 18 | R+7 | 18.45 | 4.99 |
| Lumican | P51884 | LUM | SLEYLDLSFNQIAR | 18 | Before SF | 510.32 | 90.29 |
|  |  |  |  | 18 | R+1 * | 446.93 | 74.35 |
|  |  |  |  | 18 | R+7 ** | 528.23 | 66.49 |
| Mannan-binding lectin serine protease 1 | P48740 | MASP1 | APGELEHGLITFSTR | 18 | Before SF | 67.39 | 11.31 |
|  |  |  |  | 18 | R+1 | 59.55 | 13.09 |
|  |  |  |  | 18 | R+7 * | 56.31 | 12.85 |
| Mannan-binding lectin serine protease 2 | O00187 | MASP2 | AGYVLHR | 18 | Before SF | 27.20 | 12.50 |
|  |  |  |  | 18 | R+1 | 25.82 | 7.00 |
|  |  |  |  | 18 | R+7 | 26.54 | 6.32 |
| MRNA for apolipoprotein E | Q6LBZ1 |  | AATVGSLAGQPLQER | 18 | Before SF | 677.45 | 200.20 |
|  |  |  |  | 18 | R+1 | 790.80 | 225.27 |
|  |  |  |  | 18 | R+7 | 778.84 | 183.07 |
| Myeloblastin | P24158 | PRTN3 | LVNVVLGAHNVR | 18 | Before SF | 0.99 | 1.21 |
|  |  |  |  | 18 | R+1 | 0.82 | 0.34 |
|  |  |  |  | 18 | R+7 | 0.67 | 0.15 |
| Neuropilin-2 | O60462 | NRP2 | ALQVVR | 18 | Before SF | 42.71 | 6.83 |
|  |  |  |  | 18 | R+1 | 41.06 | 5.51 |
|  |  |  |  | 18 | R+7 ** | 46.79 | 7.89 |
| Phosphatidylcholine-sterol acyltransferase | P04180 | LCAT | SSGLVSNAPGVQIR | 18 | Before SF | 34.54 | 13.53 |
|  |  |  |  | 18 | R+1 | 31.66 | 8.92 |
|  |  |  |  | 18 | R+7 | 31.92 | 7.16 |
| Phospholipid transfer protein | P55058 | PLTP | FLEQELETITIPDLR | 18 | Before SF | 284.86 | 32.19 |
|  |  |  |  | 18 | R+1 | 274.71 | 22.91 |
|  |  |  |  | 18 | R+7 | 287.47 | 26.09 |
| Pigment epithelium-derived factor | P36955 | SERPINF1 | LAAAVSNFGYDLYR | 18 | Before SF | 226.86 | 38.43 |
|  |  |  |  | 18 | R+1 | 208.97 | 48.81 |
|  |  |  |  | 18 | R+7 | 214.16 | 32.36 |
| Plasma protease C1 inhibitor | P05155 | SERPING1 | FQPTLLTLPR | 18 | Before SF | 260.80 | 134.25 |
|  |  |  |  | 18 | R+1 | 218.53 | 63.56 |
|  |  |  |  | 18 | R+7 | 244.96 | 66.47 |
| Plasma serine protease inhibitor | P05154 | SERPINA5 | FSIEGSYQLEK | 18 | Before SF | 89.89 | 26.45 |
|  |  |  |  | 18 | R+1 | 91.84 | 13.65 |
|  |  |  |  | 18 | R+7 * | 104.66 | 22.32 |
| Plasminogen | P00747 | PLG | LFLEPTR | 18 | Before SF | 56.00 | 14.99 |
|  |  |  |  | 18 | R+1 | 52.12 | 10.29 |
|  |  |  |  | 18 | R+7 | 53.33 | 8.66 |
| Protein AMBP | P02760 | AMBP | HHGPTITAK | 18 | Before SF | 926.03 | 328.32 |
|  |  |  |  | 18 | R+1 | 1007.59 | 222.46 |
|  |  |  |  | 18 | R+7 | 1080.54 | 223.87 |
| Protein S100-A9 | P06702 | S100A9 | DLQNFLK. | 9 | Before SF | 2.72 | 1.30 |
|  |  |  |  | 14 | R+1 * | 6.16 | 4.52 |
|  |  |  |  | 16 | R+7 ** | 3.81 | 1.97 |
| Protein Z-dependent protease inhibitor | Q9UK55 | SERPINA10 | LILVDYILFK | 18 | Before SF | 13.52 | 3.34 |
|  |  |  |  | 18 | R+1 | 12.93 | 2.63 |
|  |  |  |  | 18 | R+7 | 13.00 | 2.43 |
| Prothrombin | P00734 | F2 | ETAASLLQAGYK | 18 | Before SF | 22.61 | 12.47 |
|  |  |  |  | 18 | R+1 | 19.04 | 5.88 |
|  |  |  |  | 18 | R+7 | 19.49 | 4.72 |
| Retinol-binding protein 4 | P02753 | RBP4 | YWGVASFLQK | 18 | Before SF | 691.65 | 167.82 |
|  |  |  |  | 18 | R+1 | 645.57 | 130.05 |
|  |  |  |  | 18 | R+7 | 671.44 | 114.12 |
| Serotransferrin | P02787 | TF | DGAGDVAFVK | 18 | Before SF | 9743.29 | 2168.44 |
|  |  |  |  | 18 | R+1 * | 8235.38 | 1746.12 |
|  |  |  |  | 18 | R+7 * | 8343.29 | 1485.16 |
| Serum albumin | P02768 | ALB | AEFAEVSK | 18 | Before SF | 598108.98 | 129395.27 |
|  |  |  |  | 18 | R+1 | 543316.87 | 88046.94 |
|  |  |  |  | 18 | R+7 | 552478.58 | 100422.61 |
| Serum amyloid A-4 protein | P35542 | SAA4 | GPGGVWAAK | 18 | Before SF | 404.54 | 94.62 |
|  |  |  |  | 18 | R+1 | 402.16 | 128.80 |
|  |  |  |  | 18 | R+7 | 354.09 | 91.60 |
| Serum paraoxonase/arylesterase 1 | P27169 | PON1 | IFFYDSENPPASEVLR | 18 | Before SF | 1396.51 | 331.83 |
|  |  |  |  | 18 | R+1 | 1358.31 | 262.38 |
|  |  |  |  | 18 | R+7 | 1322.63 | 278.37 |
| Serum paraoxonase/lactonase 3 | Q15166 | PON3 | LLNYNPEDPPGSEVLR | 18 | Before SF | 19.95 | 5.13 |
|  |  |  |  | 18 | R+1 | 20.17 | 3.73 |
|  |  |  |  | 18 | R+7 | 18.77 | 4.60 |
| Sex hormone-binding globulin | P04278 | SHBG | IALGGLLFPASNLR | 18 | Before SF | 27.62 | 10.08 |
|  |  |  |  | 18 | R+1 | 24.92 | 7.34 |
|  |  |  |  | 18 | R+7 | 25.16 | 7.28 |
| Thyroxine-binding globulin | P05543 | SERPINA7 | AVLHIGEK | 18 | Before SF | 166.86 | 45.18 |
|  |  |  |  | 18 | R+1 | 173.56 | 53.68 |
|  |  |  |  | 18 | R+7 | 171.63 | 51.08 |
| Transthyretin | P02766 | TTR | ALGISPFHEHAEVVFTANDSGPR | 18 | Before SF | 26010.57 | 2797.80 |
|  |  |  |  | 18 | R+1 | 24649.54 | 2775.77 |
|  |  |  |  | 18 | R+7 * | 24047.41 | 3026.42 |
| Vasorin | Q6EMK4 | VASN | YLQGSSVQLR | 18 | Before SF | 15.73 | 4.14 |
|  |  |  |  | 18 | R+1 | 15.34 | 3.88 |
|  |  |  |  | 18 | R+7 | 16.06 | 3.42 |
| Vitamin D-binding protein | P02774 | GC | THLPEVFLSK | 18 | Before SF | 3222.07 | 617.49 |
|  |  |  |  | 18 | R+1 | 3301.95 | 669.23 |
|  |  |  |  | 18 | R+7 | 3306.51 | 580.94 |
| Vitamin K-dependent protein C | P04070 | PROC | LGEYDLR | 18 | Before SF | 40.35 | 9.68 |
|  |  |  |  | 18 | R+1 | 40.56 | 8.99 |
|  |  |  |  | 18 | R+7 | 41.49 | 8.67 |
| Vitamin K-dependent protein S | P07225 | PROS1 | VYFAGFPR | 18 | Before SF | 346.31 | 77.22 |
|  |  |  |  | 18 | R+1 | 343.67 | 68.21 |
|  |  |  |  | 18 | R+7 | 350.76 | 73.29 |
| Vitronectin | P04004 | VTN | VDTVDPPYPR | 18 | Before SF | 325.05 | 71.92 |
|  |  |  |  | 18 | R+1 | 326.41 | 87.79 |
|  |  |  |  | 18 | R+7 | 352.07 | 81.45 |
| Zinc-alpha-2-glycoprotein | P25311 | AZGP1 | EIPAWVPFDPAAQITK | 18 | Before SF | 105.47 | 27.79 |
|  |  |  |  | 18 | R+1 | 95.27 | 18.51 |
|  |  |  |  | 18 | R+7 | 95.12 | 13.03 |
|  |  |  |  |  |  |  |  |
|  |  |  |  |  | *** - p<0.05 vs Before SF** | | |
|  |  |  |  |  |  | | |
|  |  |  |  |  | **** - p<0.05 R+7 vs R+1** | | |
